# Ecological interactions in glacier environments: a review of studies on a model Alpine glacier

**DOI:** 10.1111/brv.13138

**Published:** 2024-09-09

**Authors:** Arianna Crosta, Barbara Valle, Marco Caccianiga, Mauro Gobbi, Francesco Gentile Ficetola, Francesca Pittino, Andrea Franzetti, Roberto Sergio Azzoni, Valeria Lencioni, Antonella Senese, Luca Corlatti, Jakub Buda, Ewa Poniecka, Tereza Novotná Jaroměřská, Krzysztof Zawierucha, Roberto Ambrosini

**Affiliations:** ^1^ Department of Environmental Science and Policy University of Milan via Celoria 26 Milan 20133 Italy; ^2^ Department of Life Sciences Università degli Studi di Siena Via A. Moro 2 Siena 53100 Italy; ^3^ NBFC, National Biodiversity Future Center Piazza Marina, 61 Palermo 90133 Italy; ^4^ Department of Bioscience University of Milan via Celoria 26 Milan 20133 Italy; ^5^ Climate and Ecology Unit, Research and Museum Collections Office MUSE‐Science Museum Corso del Lavoro e della Scienza 3 Trento 38122 Italy; ^6^ Department of Earth and Environmental Sciences University of Milano‐Bicocca Piazza della Scienza 1 Milan 20126 Italy; ^7^ Department of Earth Sciences ‘A. Desio’ University of Milan via Mangiagalli 34 Milan 20133 Italy; ^8^ ERSAF – Direzione Parco Stelvio via De Simoni 42 Bormio (SO) 23032 Italy; ^9^ Chair of Wildlife Ecology and Management University of Freiburg Tennenbacher Str. 4 Freiburg 79106 Germany; ^10^ Department of Animal Taxonomy and Ecology, Faculty of Biology Adam Mickiewicz University Uniwersytetu Poznańskiego 6 Poznań 61‐614 Poland; ^11^ Laboratory of RNA Biology – ERA Chairs Group International Institute of Molecular and Cell Biology in Warsaw 4 Ks. Trojdena Street Warsaw 02‐109 Poland; ^12^ Department of Ecology, Faculty of Science Charles University Viničná 7 Prague 2 CZ‐12844 Czech Republic; ^13^ Institute of Soil Biology and Biogeochemistry Biology Centre CAS České Budějovice 37005 Czech Republic

**Keywords:** glacier ecology, trophic webs, supraglacial ecosystem, cryophiles, ecological interactions, glacial ecosystems

## Abstract

Glaciers host a variety of cold‐adapted taxa, many of which have not yet been described. Interactions among glacier organisms are even less clear. Understanding ecological interactions is crucial to unravelling the functioning of glacier ecosystems, particularly in light of current glacier retreat. Through a review of the existing literature, we aim to provide a first overview of the biodiversity, primary production, trophic networks, and matter flow of a glacier ecosystem. We use the Forni Glacier (Central Italian Alps) – one of the best studied alpine glaciers in the world – as a model system for our literature review and integrate additional original data. We reveal the importance of allochthonous organic matter inputs, of Cyanobacteria and eukaryotic green algae in primary production, and the key role of springtails (*Vertagopus glacialis*) on the glacier surface in sustaining populations of two apex terrestrial predators: *Nebria castanea* (Coleoptera: Carabidae) and *Pardosa saturatior* (Araneae: Lycosidae). The cryophilic tardigrade *Cryobiotus klebelsbergi* is the apex consumer in cryoconite holes. This short food web highlights the fragility of nodes represented by invertebrates, contrasting with structured microbial communities in all glacier habitats. Although further research is necessary to quantify the ecological interactions of glacier organisms, this review summarises and integrates existing knowledge about the ecological processes on alpine glaciers and supports the importance of glacier‐adapted organisms in providing ecosystem services.

## I. INTRODUCTION

Habitat loss and climate change are well‐known major threats to biodiversity. Recent scientific literature emphasises how ongoing glacier retreat, triggered by climate warming, is affecting biodiversity along glacier forelands (e.g. Bosson *et al*., 2023; Ficetola *et al*., 2021). However, little is known about the biodiversity of glaciers, i.e. about the organisms living on, in, and under glaciers (Zawierucha & Shain, 2019; Stibal *et al*., 2020), and published information is not equally available for all glacier taxa. Glaciers and ice sheets have been recognised as a biome in their own right (Anesio & Laybourn‐Parry, 2012), and in Europe are protected by the Habitat Directive under the habitat type ‘Permanent Glaciers‐Code 8340’ (see Gobbi *et al*., 2021).

All glaciers are characterised by the presence of three environments: supraglacial, englacial and subglacial (Hodson *et al*., 2008), each comprising a variety of habitats hosting different communities. The supraglacial environment occurs at the ice–atmosphere interface and receives debris, dust, microorganisms, spores, pollen, and contaminants from atmospheric deposition – originating from both long‐range transport and the adjacent habitats. The surface is characterised by high irradiation, temperature cycles and is often open to the atmosphere, allowing gas exchange. However, dark and anoxic conditions can be found in some microhabitats (e.g. lower cryoconite layers; Buda *et al*., 2022). Liquid water is often available during the ablation season, but also contributes to washing out nutrients and organic matter (OM) into meltwaters (Hodson *et al*., 2008). According to current knowledge, the supraglacial environment is the most biologically productive and species rich among glacier environments. Cryoconite holes – water pools with fine debris at the bottom that form on the ice surface due to differential melting – are hotspots of microbiological activity on the ice (Hodson *et al*., 2008; Cook *et al*., 2016; Smith *et al*., 2016). Interaction of minerals and glacial Cyanobacteria leads to the formation of cryoconite granules (e.g. Takeuchi, Nishiyama & Li, 2010), which play pivotal roles in biogeochemical cycles, storing OM and reducing albedo (Segawa *et al*., 2020; Wejnerowski *et al*., 2023).

Depending on the thermal regime of the glacier, the englacial environment hosts viable communities or is limited to storing inactive microorganisms or genetic material (Makowska‐Zawierucha *et al*., 2022; Varliero *et al*., 2021). Finally, the subglacial environment lies below the bulk ice at the interface with the bedrock (Hodson *et al*., 2008). In the englacial and subglacial environments, light availability and oxygen content decrease with ice thickness. Debris content, the presence of liquid water, and the supply of nutrients and organismal seed inputs from the glacier surface depend on parameters as lithology, temperature, and seasonality (Hodson *et al*., 2008). Stress and strain due to ice movement and bedrock irregularity become important factors and, in some cases, can negatively affect the occurrence and distribution of organisms. Due to logistical and technical constraints, knowledge about subglacial communities is limited, but they are generally believed to be dominated by anaerobic microbial processes (Achberger *et al*., 2017).

Glacial organisms include (*i*) microbes – bacteria [e.g. Alpha‐ and Betaproteobacteria, Cyanobacteria, and Actinobacteria (e.g. Franzetti *et al*., 2017*a*; Pittino *et al*., 2018)], filamentous fungi and yeasts [e.g. Dothideomycetes, Microbotryomycetes, and Pleosporales (e.g. Dhume, Tsuji & Singh, 2022; Edwards *et al*., 2013; Turchetti *et al*., 2008)], archaea (e.g. Pittino *et al*., 2023*b*), and protozoans [e.g. algae and ciliates (Hoham & Remias, 2020; Zawierucha *et al*., 2022)]; (*ii*) microinvertebrates, such as tardigrades, rotifers, nematodes and platyhelminths (Zawierucha *et al*., 2019*a*; Shain *et al*., 2021); (*iii*) macroinvertebrates, such as annelids (Shain *et al*., 2001; Liang, 1979), and arthropods [mites and springtails (Buda *et al*., 2020*a*; Valle *et al*., 2022, 2024); chironomids (Boothroyd & Cranston, 1999; Kohshima, 1985; Gobbi & Lencioni, 2021); plecopterans (Vera, Zuñiga‐Reinoso & Muñoz‐Escobar, 2012); copepods (Shain *et al*., 2021); beetles and spiders (Gobbi & Lencioni, 2021)]; and (*iv*) bryophytes and vascular plants (e.g. Tampucci *et al*., 2017). Some of these glacier‐adapted macroinvertebrates may also sustain organisms at higher trophic levels. For example, ice worms (*Mesenchytraeus solifugus*) on glaciers in the Pacific Northwest of the USA are a regular food source for nesting grey‐crowned rosy finches (*Leucosticte tephrocotis*), and several other species (Hotaling *et al*., 2020). Hotaling *et al*. (2020) suggested that, close to glacierised areas, highly mobile species such as birds could take advantage of glacier subsidies by crossing habitat boundaries. Indeed, some bird species show a preference for glaciers and snow fields (Brooks *et al*., 2023). Although this evidence highlights a more important ecological role of glaciers than expected in the past, little information is available on the trophic networks and other ecological interactions (e.g. facilitation, competition) within glacier environments, representing an important gap in our knowledge of these ecosystems.

Most of the available literature focusses on the biodiversity of polar glaciers, with studies on most alpine glaciers lagging behind (Cook *et al*., 2016; Zawierucha & Shain, 2019), although Alpine debris‐covered glaciers are an exception (Gobbi, Isaia & De Bernardi, 2011; Gobbi *et al*., 2017; Tampucci *et al*., 2016, 2017; Valle *et al*., 2020, 2022). In these glaciers, most of the ablation zone is covered by thick debris. Alpine glaciers mostly occur at high altitudes, which favours greater fragmentation and isolation of the communities that inhabit them. For instance, communities of beetles and spiders were found to be more similar along the foreland of an Alpine glacier and along adjacent forelands than they were to those at distant (>30 km) sites (Gobbi & Brambilla, 2016). Alpine glaciers are on average smaller than polar glaciers and therefore more likely to disappear in the near future, implying that alpine cold‐adapted and cryophilic fauna is at a high risk of extinction (e.g. Bosson *et al*., 2023; Panza & Gobbi, 2022). As suggested by Gobbi *et al*. (2021), these taxa should be considered threatened given their areal reduction coupled with small population sizes, patchy and/or restricted distributions, low dispersal potential, stenothermy, and lack of refugia. Furthermore, many are Alpine endemites or steno‐endemites (e.g. Valle *et al*., 2024), highlighting the urgency to study the biodiversity of alpine glaciers.

By qualitatively summarising the available information on biodiversity and trophic interactions on one of the most studied Alpine glaciers – the Forni Glacier, Central Italian Alps – and combining it with new data, this study aims to fill knowledge gaps by providing a general overview of the ecological interactions in glacier environments and a description of the biodiversity of an alpine glacier.

The Forni is an ideal glacier for such a study, as almost all the taxa present on it have been studied (Table 1; see online Supporting Information, Table S1). This is one of the very few alpine glaciers in the world with sufficient multidisciplinary studies available to reconstruct, at least qualitatively, the trophic network of its supraglacial habitats. Only a few other glaciers have been regularly investigated in the context of biology and ecology, such as the Ecology Glacier in Antarctica (e.g. Buda *et al*., 2020*b*; Mieczan *et al*., 2013) and the Ürümqi Glacier in Central Asia (e.g. Zawierucha *et al*., 2018; Takeuchi *et al*., 2010). However, the Forni Glacier is the only one for which there has been regular monitoring of biota spanning more than a decade (e.g. Gobbi, Fontaneto & De Bernardi, 2006*b*). Moreover, it is among the few alpine glaciers with data available on the biogeochemistry of supraglacial sediments, meteorological conditions, ice dynamics, geological setting, and primary succession along the foreland (e.g. Rozwalak *et al*., 2022; Senese *et al*., 2018, 2020; Urbini *et al*., 2017; Azzoni *et al*., 2018; Montrasio *et al*., 2012; Gobbi *et al*., 2006*a*, 2007; Franzetti *et al*., 2020).

**Table 1:** List of publications from which information on the biodiversity and trophic interactions on the surface of the Forni Glacier was obtained. COI, cytochrome oxidase subunit 1; eDNA, environmental DNA; ITS1, internal transcribed spacer 1; rDNA, ribosomal DNA.

Reference	Method	Habitat 1	Habitat 2	Habitat 3	Focal taxon 1	Focal taxon 2
Azzoni *et al*. (2018)	16S rDNA sequencing	Snow			Bacteria	
Buda *et al*. (2020*a*)	Visual transects and counts	Supraglacial stones	Medial moraine		Springtails	
Buzzini *et al*. (2005)	Cultures, visual, biochemical identification	Subglacial stream	Supraglacial stream		Yeasts	
Franzetti *et al*. (2016)	16S, metagenomic sequencing	Cryoconite holes			Bacteria	
Franzetti *et al*. (2017*a*)	16S rDNA sequencing	Cryoconite	Lateral, medial moraines	Dirt cone	Bacteria	
Franzetti *et al*. (2017*b*)	16S rDNA sequencing	Cryoconite holes			Bacteria	
Gobbi *et al*. (2006*a*)	Pitfall traps, visual identification	Ice	Moraine		Arthropods	
Gobbi *et al*. (2007)	Pitfall traps, visual identification	Lateral, medial moraines	Supraglacial debris	Ice	Arthropods	
Novotná Jaroměřská *et al*. (2023)	Stable isotopes (C, N)	Cryoconite holes	Ice	Supraglacial debris	Tardigrades	Springtails
Pelfini & Gobbi, (2005)	Pitfall traps, visual identification	Ice	Supraglacial debris	Lateral moraine	Arthropods	
Pittino *et al*. (2018)	16S rDNA sequencing	Cryoconite holes			Bacteria	
Pittino *et al*. (2021)	RNA retrotranscription	Cryoconite holes			Bacteria	
Pittino *et al*. (2023*b*)	16S genes and transcripts, shotgun metatranscriptomics	Cryoconite holes			Bacteria	
Rozwalak *et al*. (2022)	16S rDNA sequencing	Cryoconite holes			Cyanobacteria	Algae
Turchetti *et al*. (2008)	Cultures, 26S rDNA sequencing	Supraglacial debris	Subglacial debris	Englacial	Yeasts	
Zawierucha *et al*. (2019*a*)	Aeroplankton traps, visual identification, COI sequencing	Cryoconite holes	Dirt cone, medial moraine		Tardigrades, rotifers	Invertebrate aeroplankton
Zawierucha *et al*. (2019*b*)	9 subsamples per 3 holes, visual identification	Cryoconite holes			Invertebrates	
Zawierucha *et al*. (2021)	18S rDNA in eDNA	Cryoconite holes			Invertebrates	
Zawierucha *et al*. (2022)	18S, 16S, ITS1 sequencing	Cryoconite holes			Tardigrades	Bacteria and microeukaryotes

Trophic networks are a representation of feeding interactions in an ecosystem, where the components (species) are connected by binary links, and enable the representation and analysis of the trophic structure and functioning of an ecosystem. Thus, they provide an important basis to understand an ecosystem's possible responses to changes in resource availability, and the ecological services that taxa, or groups of taxa, provide (Schwartz *et al*., 2000; Wagg *et al*., 2019). Therefore, information about trophic webs is crucial to assess the possible responses of glacier communities to the rapid retreat of their habitats, and the potential consequences of glacier disappearance on neighbouring ecosystems. As glaciers act as cold traps for contaminants (Grannas *et al*., 2013) and have the potential for contaminant degradation (e.g. Ferrario *et al*., 2017), reconstructing trophic webs on their surface can also help assessments of the effects and fate of these substances (Lencioni *et al*., 2023*b*; Pittino *et al*., 2023*a*).

## II. METHODS

### (1) Model system

**Fig. 1:**
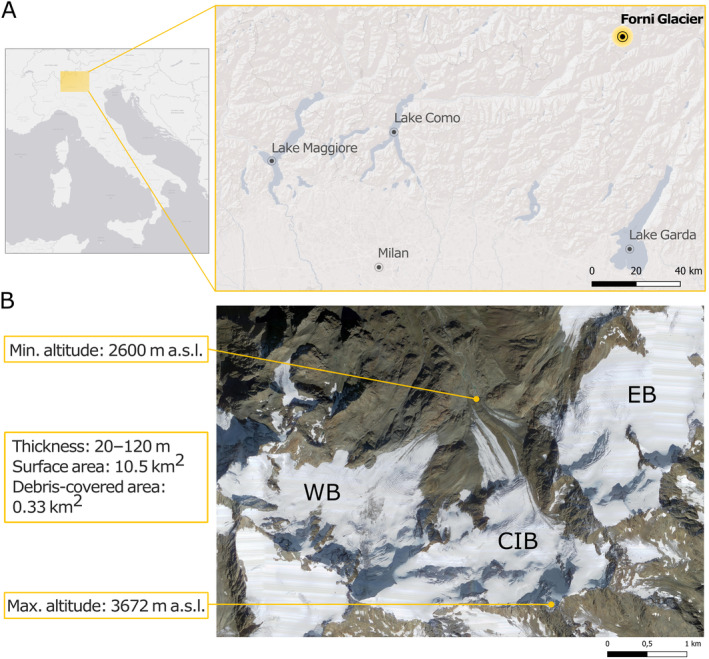
(A) Geographical location of the Forni Glacier. (B) Aerial view of the glacier. CIB, Central Ice Body, EB, Eastern Basin; WB, Western Basin.

The Forni Glacier (46° 23′ 32″ N, 10° 35′ 28″ E, Fig. 1) is located within the Stelvio National Park in the Central Italian Alps and within a Special Area of Conservation (SAC, code IT2040014). It is a valley glacier, fed in the past by three accumulation basins (Fig. 1B). The western and eastern basins separated from the central ice body in 2013 and 2015 respectively, transforming the Forni Glacier into a valley glacier and two lateral cirque glaciers. The glacier is north‐facing and extends between approximately 2600 and 3672 metres above sea level (m a.s.l.) (Fig. 1B), with a total surface area of 10.49 km^2^ in 2016 for the three ice bodies as a whole (Paul *et al*., 2020). It is classified as a temperate glacier, implying a faster flow of the ice mass (40–45 m/year in the upper sectors and 10–15 m/year at the terminus; Urbini *et al*., 2017) and abundant meltwater excavating bédières (small supraglacial streams), moulins, and other englacial or subglacial drainage systems. The surrounding rock formations are micaschists (Montrasio *et al*., 2012). The strong weathering occurring at high‐altitude bare‐rock sites implies a large availability for biological activity of silicates, bivalent cations [e.g. calcium (Ca^2+^), magnesium (Mg^2+^), iron (Fe^2+^), and manganese (Mn^2+^)] and trivalent cations [e.g. aluminium (Al^3+^), iron (Fe^3+^), and chromium (Cr^3+^)].

As one of the largest Italian glaciers and due to its accessibility, the Forni Glacier has iconographic documentation dating back to the mid‐19th century. Its meltwaters are used to power hydroelectric plants, and the Forni Glacier has strong socio‐cultural, scientific, and economic value. For these reasons, the glaciological, biological, and ecological processes of this glacier have been subject to intensive study.

### (2) Literature search

A literature search was conducted in *Web of Science*, *Google Scholar*, and *Scopus* using the key words: ‘Ghiacciaio dei Forni’/‘Forni Glacier’/‘glacier’ AND ‘ecology’; ‘Ghiacciaio dei Forni’/‘Forni Glacier’/‘glacier’ AND ‘biodiversity’; ‘Ghiacciaio dei Forni’/‘Forni Glacier’/‘glacier’ AND ‘vegetation’; ‘Ghiacciaio dei Forni’/‘Forni Glacier’/‘glacier’ AND ‘fauna’. On September 19th, 2023, this search returned 634 papers. After exclusion of papers not specifically focussed on the Forni Glacier, we retained a total of 19 papers. Seven of these focussed on bacterial communities, one on cyanobacteria and algae, four on springtails and other arthropods, two on yeasts, four investigated invertebrates in cryoconite holes (e.g. tardigrades, rotifers), and one studied the trophic levels of tardigrades and springtails (Table 1).

From these papers, we extracted information on the presence or absence of taxa in different glacier habitats (e.g. cryoconite holes, surface ice, moraine, dirt cones) to assess the biodiversity of the Forni. We then identified trophic interactions already described in the literature. Whenever data on trophic interactions were unavailable for the Forni, we searched for such information in studies about other glaciers, prioritising those that were geographically close. We thus included: (*i*) diet preferences of predators on the Forni, which were investigated in proglacial plains at sites in the Alps by Sint *et al*. (2019); (*ii*) information about algae from nearby sites (Di Mauro *et al*., 2020), laboratory settings (Perini *et al*., 2023), and other alpine areas (Brown, Olson & Jumpponen, 2015) to determine the roles of snow algae detected on the Forni Glacier, but not investigated there; (*iii*) enzymatic activity for one yeast strain detected on the Forni, whose activity has not been studied there, but was available for Antarctic or Himalayan sites (Martorell *et al*., 2017; Sanyal *et al*., 2020), although the extent to which the strains have equivalent activity at these sites remains unclear.

In the available literature, the biodiversity of the Forni was investigated by the flotation methods, pitfall trapping, and hand searching (for arthropods), DNA metabarcoding, visual microscopic identification, and cytochrome oxidase subunit 1 (COI) sequencing. Different habitats (e.g. medial moraine, cryoconite holes, and sub‐ and englacial habitats for yeasts) were investigated. The trophic levels of Collembola and Tardigrada were investigated using stable isotope analysis of δ^13^C and δ^15^N (Novotná Jaroměřská *et al*., 2023) and environmental DNA (eDNA) (Zawierucha *et al*., 2022). Further details on the methods used and the habitats described are provided in Table 1.

### (3) Original data

Previously unpublished information was used to fill some gaps in the literature. Bryophytes were qualitatively sampled from the medial moraine of Forni and a few isolated boulders on the bare ice, then air‐dried in paper bags for preservation. After rehydration with water, identification to genus level was performed using dichotomic keys from Cortini Pedrotti (2001, 2005) under a stereomicroscope. Information on the occurrence of springtail species was obtained from qualitative sampling on the medial moraines using the flotation method (Valle *et al*., 2022), and under isolated boulders on bare ice by hand catching. Specimens were preserved in 96% alcohol and then identified to species level using taxonomic keys from Gisin (1960), Potapov (2001) and Valle *et al*. (2024). Data obtained from these two qualitative samplings are included in the checklist of species detected on the Forni Glacier (Table S1). Observations of vertebrate species were opportunistically collected during fieldwork. eDNA data from cryoconite holes on the Forni Glacier [see Zawierucha *et al*. (2022) for methodological details], were used to confirm the presence of organisms and trophic interactions. Information on eDNA abundance is available in Tables S2 and S3, and Appendix S1. To assess the structure of eukaryotic communities, we also sequenced the V9 region of the 18S rDNA gene with Ion Torrent (Life Technologies). DNA was extracted with FastDNA SPIN Kit for Soil (MP Biomedicals) from 10 wet cryoconite samples (about 0.5 ml each). For library preparation and sequencing we referred to Zawierucha *et al*. (2022). Sequences were assembled into amplicon sequence variants (ASVs) using the DADA2 package (Callahan *et al*., 2016). Taxonomy was assigned using the SILVA database (v128.18 s.99; Quast *et al*., 2013) with full classification for confidence values equal to or higher than 0.8. ASVs not classified to family level, and those with only a few reads are not considered in the description of the possible trophic structure. Abundance data are available in Table S4. The radiative input during the snow‐free period (i.e. July, August and September) on the Forni Glacier was calculated using solar radiation data recorded by the automatic weather station on the Forni ablation area [AWS1 Forni, active since September 2005 (see e.g. Senese *et al*., 2018, 2020)]. All new data presented here are available at: https://doi.org/10.13130/RD_UNIMI/9PPBAB

## III. RESULTS

### (1) Supraglacial environment

The most widespread supraglacial habitat on the Forni Glacier is the bare ice surface. On the ablation tongue, areas free from debris (coarse, fine or cryoconite) were estimated at 73.3% (0.65 km^2^) in 2003 and 51.9% (0.35 km^2^) in 2015 (Azzoni *et al*., 2018). However, little information is available regarding the communities on the bare ice surface of the Forni. By contrast, cryoconite holes have been extensively studied over a longer period (e.g. Pittino *et al*., 2018). On the Forni, cryoconite is in the form of dark irregular granules, repeatedly observed over many years, with a mean ± SD size of 0.54 ± 0.20 mm (Rozwalak *et al*., 2022). The OM content varies among years and with location of cryoconite holes, and generally increases during the ablation season (Pittino *et al*., 2018), ranging between 5% and 55% (Rozwalak *et al*., 2022). As on other temperate glaciers (Takeuchi *et al*., 2000), cryoconite holes on the Forni do not last throughout the whole ablation season. An important feature of temperate glaciers is the high abundance of running water during the melting season. Meltwaters flow in bédières on the ice surface: their flow contributes to the dispersal of cryoconite, microbes, and microinvertebrates, and to leaching of nutrients off the glacier (Hodson *et al*., 2008). Consequently, the various habitats in the supraglacial zone of the Forni Glacier are interconnected.

Few studies have investigated colonisation by organisms through atmospheric deposition on the Forni. Zawierucha *et al*. (2019*a*) conducted a short‐term experiment with aeroplankton traps and found windblown, non‐glacial mites on the glacier surface. Snowfalls can also efficiently precipitate bacteria from the atmosphere and provide either a source for glacier communities or additional allochthonous OM. However, on the Forni the snow bacterial community has been studied only in seasonal snow and no information is available for fresh snow. In seasonal snow, the orders *Cytophagales* and *Burkholderiales* dominate (Azzoni *et al*., 2018). Abundant percolation was described during sample collection, suggesting that cells and smaller organisms may be transported vertically as the snow melts and reach the ice surface (Azzoni *et al*., 2018).

Trophic levels and potential interactions among organisms were reconstructed only for the well‐studied supraglacial environment and include: (*i*) contribution of allochthonous OM; (*ii*) primary producers; (*iii*) autochthonous OM; (*iv*) decomposers; and (*v*) consumers.

#### (a) Allochthonous organic matter

We defined allochthonous OM as OM originating from organisms from habitats other than the glacier and unable to survive there. Allochthonous OM has been detected on the glacier surface by several studies. Pelfini & Gobbi (2005) found specimens belonging to the orders Diptera, Hymenoptera, Coleoptera (Elateridae: *Fleutiauxellus maritimus*), and Hemiptera (*Cinara* sp.) sampled in pitfall traps placed in coarse detritus on the glacier surface (Table S1). Collembola, Acarina, Opiliones and Tardigrada were detected in aeroplankton traps (Zawierucha *et al*., 2019*a*). Data from Zawierucha *et al*. (2019*a*) allow estimates of the average deposition rates of the cited taxa as 7.1 individuals per square metre (ind/m^2^) on the ice, and 3.8 ind/m^2^ on the medial moraine, with an average for the whole glacier of 5.5 ind/m^2^. However, more information is needed for a complete characterisation of allochthonous OM and its contributors. In cryoconite holes, we detected eDNA of Polyphaga (Coleoptera), *Apis mellifera* (Hymenoptera), and Sciaridae (Diptera) (Tables S2 and S3). All these taxa were probably transported there by upslope winds at high altitudes, where the temperature is too low for their survival as observed for other glacial environments (Coulson, Hodkinson & Webb, 2003; Gobbi *et al*., 2011; Flø & Hågvar, 2013). Similarly, leaves and seeds are often observed on the glacier surface (A. Crosta, B. Valle, R. Ambrosini & F. Pittino, personal observations) and, together with pollen, contribute to allochthonous OM. Pollen can be transported from local and distant locations and represents a source of organic phosphorus on the ice surface (Grewling *et al*., 2024). Several fungal genera linked to non‐glacial environments were also detected on the Forni: the forest fungi *Russula*, *Xerocomus*, and *Pseudotomentella* were likely transported to the glacier as fragments or spores (Zawierucha *et al*., 2022). 18S rDNA sequencing of cryoconite samples from the Forni identified abundant Blastocladiales, which are decomposers of plant material (Tables S1 and S4). eDNA analysis of the same habitat detected other soil fungi such as *Basidioascus undulatus*, and members of the families Powellomycetaceae and Lobulomycetaceae (Tables S2 and S3). Among plants, we found eDNA of *Pleurozia purpurea* (Hepaticae) and the subfamilies Ranunculoideae, Pooidae and Onagroideae, together with that of members of the subclass Pinidae – the presence of these is again plausibly due to aerial transport. However, *Poa laxa*, *P. alpina* and *Ranunculus glacialis* are three species that are frequently found close to the glacier and on debris‐covered glaciers (Tampucci *et al*., 2016), and we therefore cannot exclude that some individuals might survive on the medial moraine of the Forni, although we never observed the presence of vascular plants. eDNA and 18S analyses were conducted only in cryoconite holes, but we can assume that atmospheric deposition is likely to be relatively homogeneous over the entire ablation area of the glacier, and thus that the allochthonous OM input will be similar for the bare ice surface. Bacteria are also transported by wind from nearby and distant sources (Franzetti *et al*., 2017*a*). Not all bacterial populations are able to survive on glaciers, and may therefore serve as a source of OM. Such windblown material is an important source of OM on some arctic glaciers (e.g. Koziol *et al*., 2019). Wind‐borne allochthonous OM also includes predators, parasites, and pathogens, some of which may be able to survive on glaciers in dormant stages. For instance, eDNA analyses detected the DNA of *Vampyrella lateritia* (a predatory freshwater amoeba that can survive freezing temperatures as a cyst), *Mycrobotrium violaceum* (parasite of Caryophyllaceae), Stylocephalidae (apicomplexan parasite of insects), *Taphrina* and Taphrinomycotina (plant pathogens), *Simulium* (parasitic flies of birds and vertebrates), and *Cryptosporidium* (protozoan parasite of vertebrates) (Tables S2 and S3).

The glacier can be considered as a geomorpho‐ecological corridor, facilitating the movement of vertebrates. The following vertebrates were occasionally observed by the authors: western house martin (*Delichon urbicum*), snow finch (*Montifringilla nivalis*), red fox (*Vulpes vulpes*), ermine (*Mustela erminea*), mountain hare (*Lepus timidus*), Northern chamois (*Rupicapra rupicapra*), and Alpine ibex (*Capra ibex*). In 2009, the footprints of a brown bear (*Ursus arctos*) were observed on the snow in the accumulation area of the glacier. Vertebrates crossing the glacier produce faeces, which are an extra source of nutrients and OM, but also of potential pathogens and faecal bacteria.

#### (b) Primary producers

The daily average maximum values of radiative input calculated for the Forni are 1209.1 W/m^2^ in July, 1114.5 W/m^2^ in August and 854.3 W/m^2^ in September. This suggests that the irradiation on the glacier surface is intense under all weather conditions (i.e. from clear sky to overcast). As a result of this high light availability in supraglacial habitats, oxygenic and anoxygenic phototrophic organisms are important primary producers on the glacier surface, together with chemosynthetic microbes (Pittino *et al*., 2021). Published data for primary producers on the Forni are only available for cryoconite holes, although algae have been detected on the glacier surface (Azzoni *et al*., 2016). Algae on the ice surface of the Forni Glacier have not been taxonomically identified to date. However, ice algae communities on the Forni can be partly reconstructed from data available for other habitats.

In cryoconite of the Forni, Cyanobacteria and algae perform oxygenic photosynthesis (Pittino *et al*., 2021). For cyanobacteria, representatives of the genera *Wilmottia*, *Chroococcus*, *Phormidium*, *Leptolyngbya*, and *Pseudanabaena* have been identified (Buda *et al*., 2022; Rozwalak *et al*., 2022). Among algae, Zawierucha *et al*. (2022) detected the classes Trebouxiophyceae and Xanthophyceae, and the family Chlamydomonadaceae (*Chloromonas* sp., and *Sanguina* sp.). However, these genera of Chlamydomonadaceae are typical snow algae. *Sanguina* has been previously detected on glaciers close to the Forni, e.g. the Morteratsch Glacier, 50 km from the Forni (Di Mauro *et al*., 2020), and *Chloromonas* is present in other European mountain ranges (e.g. Procházková *et al*., 2019). Through eDNA and 18S rDNA sequencing we detected a high abundance of Mischococcales (Xanthophyceae). Rozwalak *et al*. (2022) identified the following species and genera: *Cylindrocystis brebissoni* (Ralfs) De Bary f. cryophila Kol, *Trochiscia granulata* (Reinsch) Hansg., *Klebsormidium*, *Chlorella*, *Mesotaenium*, *Trochiscia*, *Fragilaria*, and *Nitzschia*. The glacier algal genus *Mesotaenium* was detected on the Morteratsch Glacier (Di Mauro *et al*., 2020) and *Cylindrocystis* on the Greenland Ice Sheet (Yallop *et al*., 2012). The presence of these two genera, and of *Chloromonas* and *Sanguina*, indicates the potential for primary production by snow and ice algae on the Forni Glacier, but further investigations are needed. Red snow has been observed occasionally on snow patches close to the glacier. Snow algae often co‐occur with fungi (e.g. Brown *et al*., 2015; Perini *et al*., 2023), suggesting possible complex interrelationships, such as enrichment of pathogen, endophytic, and saprotrophic fungi within snow communities (Gostinčar & Gunde‐Cimerman, 2023). The growth and abundance of snow and ice algae might be modulated positively by fungi (see Perini *et al*., 2023). As *Mesotaenium* (also known as *Ancylonema alaskanum*) has been detected on the Forni, similar interactions could be taking place, although supporting evidence is still needed. Within the class Trebouxiophyceae, eDNA analysis detected the family Botryidiopsidaceae (freshwater algae also detected in Antarctica; Suh *et al*., 2017) and the freshwater alga *Hydrurus foetidus*, a cold‐adapted benthic member of Chrysophyta. *Hydrurus foetidus* is often linked to the occurrence of non‐biting midge larvae (Diptera: Chironomidae) in glacial streams (Lencioni & Rossaro, 2005). Therefore, we cannot exclude the presence of chironomids in bediérès on the surface of the Forni, especially as *Diamesa steinboecki* and *Metriocnemus eurynotus* gr. have been reported from the Brenta Dolomites 30 km away (Lencioni *et al*., 2021). eDNA analysis additionally detected *Ochromonas* sp. CCMP1899, also found in the McMurdo Dry Valleys of Antarctica (Roberts *et al*., 2004), and *Chlorolobion braunii*. Cyanobacteria and algae are active throughout the day, as is anoxygenic photosynthesis (Pittino *et al*., 2023*b*). Most aerobic anoxygenic phototrophs are affiliated with Alpha‐ and Betaproteobacteria, which are known to use OM as a substrate and light to complement energy demand (Pittino *et al*., 2021). When the activity of phototrophs decreases, such as during the night, chemosynthetic Proteobacteria are the most active taxa involved in primary production. Hydrogen oxidation then might become an important pathway to carbon fixation, with hydrogenase gene transcripts on the Forni mostly assigned to *Paracoccus* sp. and *Hydrogenophylus* sp. Both genera can exploit molecular hydrogen as an electron donor (Bardischewsky & Friedrich, 2001; Hayashi *et al*., 1999). On glaciers, molecular hydrogen may have abiotic origins, arising from reactions between water and fine siliceous debris (Telling *et al*., 2015), which is abundant on the Forni due to the surrounding rock formations. However, H_2_ may also result from fermentative and photosynthetic processes in the anoxic zone of cryoconite (Poniecka *et al*., 2018), found on average 1500 μm below the cryoconite–water interface on the Forni Glacier (Buda *et al*., 2022), or can be produced by methanogenic archaea, also present on the Forni (Pittino *et al*., 2023*b*). Other autotrophs in cryoconite holes are carbon monoxide (CO) oxidisers, which have been classified as Actinobacteria and β‐Proteobacteria (Franzetti *et al*., 2016). These bacteria could exploit CO production through photodegradation of dissolved organic matter (DOM; Franzetti *et al*., 2016).

To date, no vascular plants have been detected on the Forni except for a dicotyledon bud on the medial moraine (B. Valle & M. Caccianiga, personal communication). However, bryophytes of the genera *Grimmia*, *Racomitrium*, *Polytrichium*, *Bryum*, and *Kiaeria* (Table S1) are common on the moraine of the Forni and on isolated boulders, and constitute an additional source of OM for the supraglacial habitats. Indeed, in cryoconite holes we detected the presence of eDNA of Polytrichaceae and *Racomitrium elongatum*, besides that of small organisms inhabiting bryophytes, such as the cercozoan *Trinema enchelys* (Tables S2 and S3).

#### (c) Autochthonous organic matter

Organisms on, inside, and beneath the glacier produce OM defined as autochthonous. Components of this category include sugars and other organic compounds produced by organisms living on the glacier, and may originate from extracellular polymeric substances (EPS), faeces or faecal pellets. Cyanobacteria and algae are well known to produce EPS, which contribute to the formation of cryoconite granules (Takeuchi *et al*., 2010). Fungi are also able to produce EPS, for example the genus *Rhodotorula* produces mannan, a polysaccharide protecting cells from freeze–thaw damage and desiccation (Touchette *et al*., 2022). Such compounds can be exploited by other organisms as a nutrient source. Snow and ice algae often produce pigments with a sugar moiety that can be exploited by fungi (Perini *et al*., 2023), although this interaction has not yet been recorded on the Forni Glacier, for which there is little information about snow and ice algae. Currently, autochthonous OM produced on the Forni Glacier has not been investigated quantitatively nor qualitatively, limiting our understanding of its contribution to trophic interactions on the glacier.

#### (d) Decomposers

Allochthonous and autochthonous OM is exploited, among others, by fungi such as *Goffeauzyma* (formerly *Cryptococcus*) *gilvescens* and *Microstroma* (formerly *Rhodotorula*) *bacarum*, detected on the Forni by Turchetti *et al*. (2008). On Alpine glaciers, *G. gilvescens* showed a strong ability to hydrolyse lipophilic substrates and degrade starch and proteinaceous substrates (Turchetti *et al*., 2008). In cryoconite holes, other yeasts of the class Microbotryomycetes, and the genus *Mortierella* were detected by eDNA sequencing, together with the water mold *Saprolegnia* (Tables S2 and S3). Many yeasts detected in other habitats on the Forni Glacier (e.g. *Leucosporidium*, *Rhodotorula*, *Phenoliferia*) belong to this class. Oomycetes of the genus *Saprolegnia* are freshwater saprophytes, while *Mortierella* has been reported in the Arctic and Antarctic (Hassan *et al*., 2016). Previous studies detected ascomycetous genera, such as *Aureobasidium*, *Preussia* (in cryoconite holes; Zawierucha *et al*., 2022) and *Microstroma bacarum* (in supraglacial debris; Turchetti *et al*., 2008). All three genera have been previously isolated from cold environments (e.g. Gunde‐Cimerman *et al*., 2003; Perini *et al*., 2019). Chytridiales and Pleosporaceae, and the genus *Cladosporium* (Ascomycota) have been detected in cryoconite of the Forni Glacier by eDNA, in accordance with results from other areas of the world (e.g. Perini *et al*., 2019; Tsuji *et al*., 2022). As for *G. gilvescens*, these taxa may benefit from the input of allochthonous and autochthonous OM. In laboratory settings, a correlation between increased abundance of Chytridiomycota and increased algal mortality suggest that these fungi function either as decomposers or parasites of glacier ice algae (Perini *et al*., 2023). Notably, the role of decomposers is probably not limited to fungi. Microorganisms of cryoconite holes, such as Alpha‐ and Betaproteobacteria, and Bacteroidetes, are known to decompose OM on other glaciers (e.g. Margesin, Zacke & Schinner, 2002; Sanyal *et al*., 2018; Stibal, Šabacká & Žárský, 2012) and probably play the same role on Forni, although no specific studies have yet been carried out.

Finally, glaciers act as cold traps for contaminants such as polycyclic aromatic hydrocarbons, polychlorobiphenyls (PCBs), and pesticides (e.g. Li *et al*., 2017; Weiland‐Bräuer *et al*., 2017) and the presence of contaminants can promote the selection of bacterial strains with metabolic pathways for their degradation (Cappa *et al*., 2014). Strains capable of degrading chlorpyrifos (an organochlorine pesticide) have been detected on the Forni (Ferrario *et al*., 2017), and we suggest that some contaminants may constitute an additional carbon source for specific bacterial strains.

#### (e) Consumers

Consumers on the Forni Glacier include both protists and metazoans. While heterotrophic protists often are primary consumers, the classification of members of either group into a specific trophic consumer level is complicated. Lack of information about the diet and foraging behaviour of many of these taxa, for example in springtails, protists and tardigrades, is a key omission.

Heterotrophic microeukaryotes such as cercozoans of the families Rhogostomidae, Gymnophryidae, Euglyphidae and Cercomonadidae were detected in cryoconite holes (Zawierucha *et al*., 2022; Tables S2 and S3). Rhogostomidae, Euglyphidae and Cercomonadidae are considered omnivorous (Khanipour Roshan *et al*., 2021). Rhogostomidae feed on bacteria and algae (Dumack *et al*., 2017), Euglyphidae on Andean glaciers feed on algae and yeasts among other unidentified food content (Santibáñez *et al*., 2011), while the feeding behaviour of Gymnophyridae is poorly known. Therefore, ascribing these taxa to a precise consumer level is extremely difficult. Our eDNA data confirmed the presence of the Rhogostomidae *Capsellina* sp., Chlamydophryidae, and *Rhogostoma* sp. 1966/2, and the Heteromitidae (cercozoans) *Heteromita* sp., *Heteromita globosa* and various Bodomorpha (especially of the family Viridiraptoridae) (Tables S2 and S3). Interestingly, *Heteromita globosa* was detected in Antarctic lakes (Hawthorn & Ellis‐Evans, 1984), *Capsellina* in subglacial ecosystems in Canada (Hamilton *et al*., 2013), and Viridiraptoridae in the Pyrenées (García‐Descalzo *et al*., 2013). eDNA also detected the presence of other protists such as *Spumella* sp., which is often found in the pelagic zone of lakes (Pernthaler & Posch, 2009), and various Ciliophora (e.g. *Paramecium* and *Stichotrichia*), which are often omnivorous (Giachello *et al*., 2023).

Invertebrate consumers on the Forni Glacier strongly segregate by habitat. Cryoconite holes are dominated by cryophilic, black tardigrades (Tardigrada) of the species *Cryobiotus klebelsbergi* (Zawierucha *et al*., 2019*a*, 2021). In coarser fractions of supraglacial debris, including under stones on the ice surface, springtails (Hexapoda: Collembola) are dominant (Buda *et al*., 2020*a*), while rotifers (Rotifera) are distributed patchily (Zawierucha *et al*., 2019*a*). *Cryobiotus klebelsbergi* was found in cryoconite holes on the Forni Glacier in seven summers (2012, 2017, 2018, 2019, 2020, 2021, 2022) at densities up to 172 ind./ml (mean 48 ind./ml) (Zawierucha *et al*., 2019*a*, *b*, 2021, 2022). The frequency of this species is surprisingly high, as it was detected in 100% of the investigated cryoconite holes (Zawierucha *et al*., 2019*a*, *b*). The dry biomass of black tardigrades in cryoconite reached 75 μg/cm^2^, with an average of 12.28 μg/cm^2^ (Novotná Jaroměřská *et al*., 2023). The mean dry biomass of springtails was similar (12.68 μg/cm^2^), however, springtail biomass peaked at 151.90 μg/cm^2^ under stones (Novotná Jaroměřská *et al*., 2023). The occurrence of bdelloid rotifers in cryoconite is very low compared to tardigrades (only a few specimens among hundreds of tardigrades). However, they are more common in bryophytes on the supraglacial boulders, where they are found in around 20% of bryophytes on supraglacial stones and debris (Zawierucha *et al*., 2019*a*).

**Fig. 2:**
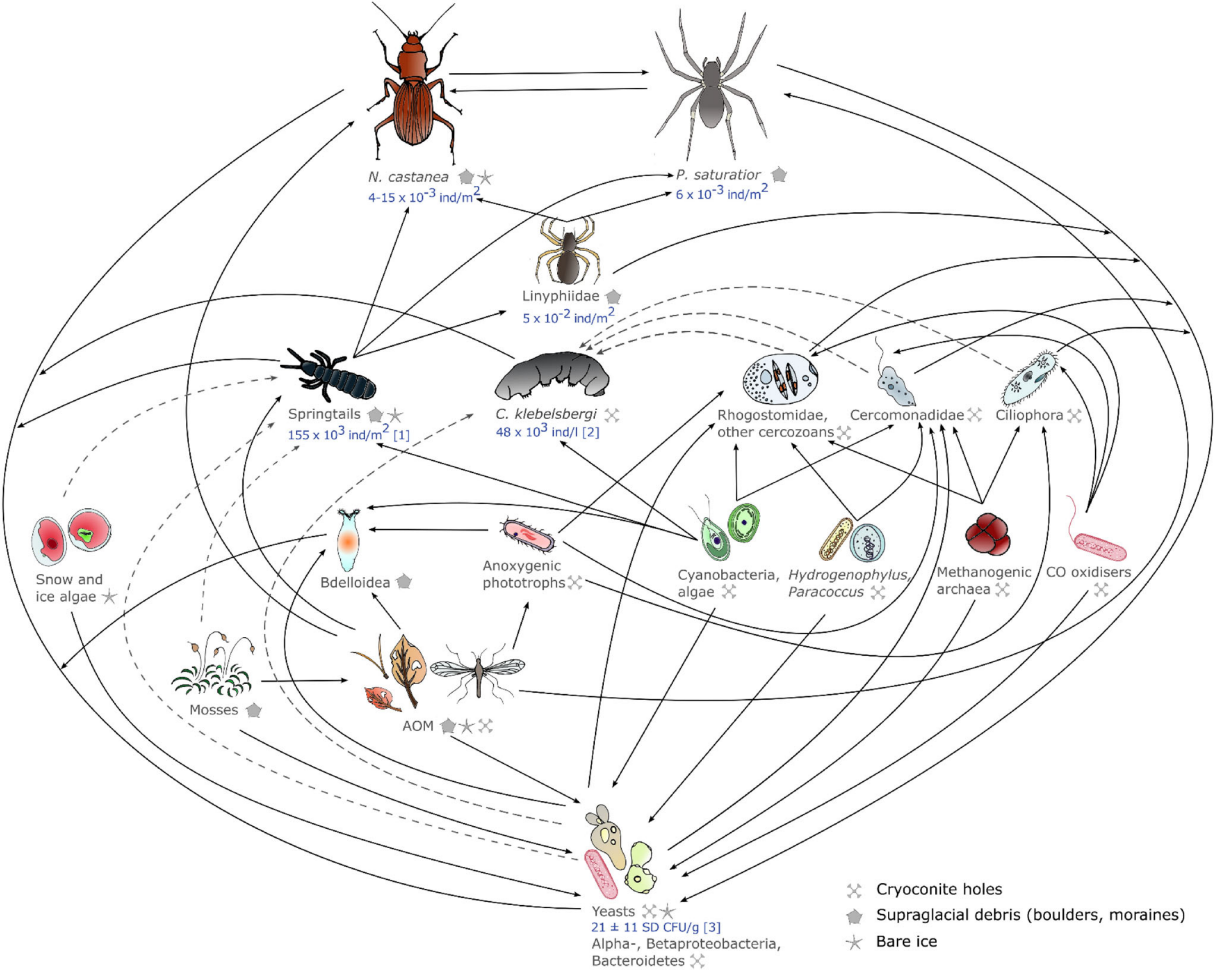
Trophic web for the supraglacial habitat of the Forni reconstructed using published information for this specific site, together with dietary studies on the same species in similar areas on other mountain ranges. Dashed lines indicate trophic interactions not described in the literature that are suggested by the authors. *N. castanea*, *Nebria castanea*; *P. saturatior*, *Pardosa saturatior*; *C. klebelsbergi*, *Cryobiotus klebelsbergi*. Abundance data are from Buda *et al*. (2020*a*) [1], Zawierucha *et al*. (2019*a*) [2], and Turchetti *et al*. (2008) [3]. Other abundance data are from our own calculations (see Section III.1.*e*).

Springtails are the dominant terrestrial invertebrates on the Forni (Buda *et al*., 2020*a*). On bare ice the dominant species is *Vertagopus glacialis*, a newly described species of ice‐dwelling springtail (Valle *et al*., 2024). Other species detected in supraglacial coarse debris are *Orchesella* cf. *alticola* and *Pachyotoma crassicauda* (Table S1). Specimens of the family Isotomidae (represented on the ice surface mainly by *Vertagopus glacialis*; Valle *et al*., 2024) are mostly found at the ice–stone interface on this glacier, and at extremely high densities (155,000 ind./m^2^; Buda *et al*., 2020*a*), suggesting they play an important role in the cycling of OM and energy flow on the glacier (Buda *et al*., 2020*a*). Ice‐dwelling springtails feed on dispersed cryoconite on the glacier surface, with gut analysis showing a high pollen intake on Austrian glaciers (Kopeszki, 1988). Accordingly, they have been classified into the herbivorous–detritivorous trophic level, similar to tardigrades, by stable isotope analyses (Novotná Jaroměřská *et al*., 2023). However, no further details about their diet and foraging behaviour are available. Whether springtails also feed opportunistically on bacteria and heterotrophic microeukaryotes in cryoconite remains unknown. This current lack of information hinders their classification into a particular consumer level. In the forelands of other Alpine glaciers, springtails represent the most important prey for both carabid beetles belonging to the genus *Nebria* and spiders of the genus *Pardosa* (Sint *et al*., 2019), and it is very likely that the same is true on the ice surface of the Forni Glacier (see below). This would suggest that springtails link the microfauna to both apex predators (Fig. 2). Current knowledge indicates that, given the low redundancy of the macrofaunal component of the ecological network of the Forni, this link is limited to springtails.

The microbial communities in cryoconite holes are grazed upon by tardigrades of the species *Cryobiotus klebelsbergi* (Zawierucha *et al*., 2022), also detected in wet supraglacial debris, and sporadically in dirt cones (Zawierucha *et al*., 2019*a*). Zawierucha *et al*. (2022) identified *Flavobacterium*, 11 other bacterial taxa, the algal classes Trebouxiophyceae, Xanthophyceae, and fungi (*Preussia* sp.) as possible food sources for *C. klebelsbergi*. Allochthonous OM probably also forms part of the diet of this species. In laboratory cultures, *C. klebelsbergi* actively feeds on *Chlorella* sp. (Zawierucha *et al*., 2023) and this same genus of eukaryotic green algae was detected on the Forni in our eDNA analysis. Tardigrades were allocated to the herbivorous–detritivorous trophic level by stable isotope analyses, as for springtails (Novotná Jaroměřská *et al*., 2023). However, potential bacterial and fungal food sources indicate that *C. klebelsbergi* may be more omnivorous than herbivorous, as would be predicted from morphological analysis alone (Zawierucha *et al*., 2022). Therefore, their ecological role in cryoconite holes can be better identified as apex consumers.

Gobbi *et al*. (2006*a*,*b*, 2007) detected two predators at the adult stage on the ice surface: the carabid beetle *Nebria castanea* (seven specimens sampled by six pitfall traps on the medial moraine and two specimens sampled by six pitfall traps on the stony debris covering the glacier over 90 days) and the wolf spider *Pardosa saturatior* (three specimens sampled by six pitfall traps on the medial moraine over 90 days, and several specimens observed in summer 2004 and 2005, on ice, supraglacial debris, and in small bediérès). We estimated the abundance of these species, assuming that each pitfall trap collects from an area of 78.5 m^2^ (in agreement with movement range estimates by Raso *et al*., 2014), to be 0.015 ind/m^2^ on the medial moraine and 0.004 ind/m^2^ on the bare ice for *N. castanea*, and 0.006 ind/m^2^ with no estimate possible for the bare ice for *P. saturatior* (Fig. 2).

During summer 2023, three adult sheet‐weaver spiders (Arachnida: Linyphiidae) were sampled by eight pitfall traps located on the median moraines. Three more individuals were sampled by hand searching under isolated stones. Following the same procedure as given above, their abundance was estimated at 0.05 ind/m^2^ on the medial moraine. Given their small size and their ability to disperse on the glacier by ballooning, their abundance may have been underestimated in previous studies based on pitfall traps, which capture larger prey more effectively.

Sint *et al*. (2019) investigated the diet composition of predators in the proglacial plains of three glaciers in the Gaisberg, Rotmoos, and Lang valleys of Tyrol. They showed that Linyphiidae feed on collembolans and are preyed upon by the larger predators *N. castanea* and *P. saturatior*. These trophic relations are conserved in later pioneer stages – when the range of available prey increases – and trophic relations are mostly influenced by predator species, rather than time since deglaciation (Sint *et al*., 2019). This suggests that they may also apply on the Forni Glacier, where prey availability is lower. Therefore, we suggest that Linyphiidae are higher‐level consumers and *N. castanea* and *P. saturatior* are best classified as apex predators.

*Nebria castanea* and *Pardosa saturatior* are ground‐dwellers. *N. castanea* is wingless (Gobbi *et al*., 2007), while *P. saturatior* does not balloon and is therefore unable to disperse by wind (Gobbi *et al*., 2011). Consequently, both have very low dispersal ability, and their persistence for multiple years on the glacier surface suggests stability of their ecological and food niches. In visual surveys during summer 2023, a larva of *Nebria castanea* was observed under stones on the glacier surface (Table S1); suggesting that this species completes its life cycle on the glacier surface. Both species are cold adapted, but *N. castanea* is nocturnal, while *P. saturatior* is diurnal (Gobbi *et al*., 2006*a*). This temporal niche segregation may be key to their co‐existence on the Forni, as it may prevent competitive exclusion. In proglacial plains in Austria, springtails were the most frequent prey for both *N. castanea* and *P. saturatior*, representing up to 50% of their diet (Sint *et al*., 2019). Similar results were obtained by previous studies in the same area, with springtails representing an important share of prey for both *N. castanea* and *Pardosa* spiders in early (0 to 7 years ice‐free) and late (12 to 20 years ice‐free) pioneer stages (Raso *et al*., 2014). This suggests that the diet of these species remains constant even among sites with different prey availability. It seems reasonable therefore, to assume that this would also be the case on the glacier surface, with the caveat that the range of prey available on the glacier surface is narrower than in proglacial plains. Given the predominance of springtails as prey shown by feeding analyses for *N. castanea* and *P. saturatior* at other sites, and the high abundance of springtails on the Forni Glacier (Buda *et al*., 2020*a*), it is highly likely that they represent the most frequent prey also on the bare ice surface. Moreover, in environments where less extraguild prey is available, intraguild predation gains importance (Lucas & Rosenheim, 2011; Polis & McCormick, 1987). In pioneer stages, spiders of the genus *Pardosa* represent important prey for both *N. castanea* and *P. saturatior*, and intraguild predation contributes up to 30% of total prey (Sint *et al*., 2019). Finally, approximately 30% of dietary composition was represented by allochthonous prey (Sint *et al*., 2019), which might also be the case on the Forni Glacier.

#### (f) Supraglacial environment: shifts in community dynamics

Glaciers are highly dynamic habitats, especially during the melting season. Due to the high melting rate (about 3–4 m water equivalent of seasonal ice melting; Senese *et al*., 2020), the depth and area of cryoconite holes increases during the ablation season (Franzetti *et al*., 2017*b*). Such changes can affect the composition of microbial communities within cryoconite holes during the ablation season (Franzetti *et al*., 2017*b*), with a pattern consistent with ecological succession – starting from colonisation by autotrophs (e.g. Cyanobacteria) and other poikilo‐tolerant taxa (e.g. Clostridiales). After the microhabitats have been altered by autotrophs and stress‐resistant taxa, and the availability of organic carbon and dissolved oxygen in the water increases, other heterotrophic populations, for example Burkholderiales and Sphingobacteriales, tend to outcompete the pioneer taxa (Franzetti *et al*., 2017*b*). In addition, because of mixing by meltwater and other physical forces, the distribution of tardigrades and probably other invertebrates at the bottom of cryoconite holes is highly heterogeneous, likely impacting productivity and biodiversity at specific locations in benthic habitats (Zawierucha *et al*., 2019*b*). Such high levels of dynamism of these communities implies that the intensity of trophic interactions may shift accordingly, and therefore a complete representation of supraglacial community dynamics may be difficult to achieve.

Likely due to seeding processes, the bacterial communities of cryoconite holes on the Forni Glacier are slightly different each melting season (Pittino *et al*., 2018). These shifts in the composition of bacterial communities across years shows that the bacterial component of the Forni Glacier's trophic web is diverse and exhibits redundancy. Consistently, numerous studies (Franzetti *et al*., 2016, 2017*a*,*b*; Azzoni *et al*., 2018; Pittino *et al*., 2018, 2021, 2023*b*) show rich autotrophic and heterotrophic communities in different glacial habitats and all light and oxygen conditions. Metabolic pathways include oxygenic and anoxygenic photosynthesis, chitin assimilation, CO oxidation, aerobic respiration, nitrification and denitrification, and sulphur and hydrogen oxidation (e.g. Pittino *et al*., 2021).

The long‐term stability of invertebrate communities is more difficult to assess, especially for taxa for which fewer data are available. *Cryobiotus klebelsbergi* was found consistently to be the dominant microinvertebrate in cryoconite holes on the Forni Glacier across a 10‐year span (Zawierucha *et al*., 2019*a*,*b*, 2021, 2022). Similarly, a few individuals of *N. castanea* were detected from 2004 to 2023 (Gobbi *et al*., 2006*a*; Table S1), indicating a stable presence on the glacier. Due to the smaller amount of data, a temporal comparison is impossible for *P. saturatior* and sheet‐weaver spiders. In general, due to the low densities of *N. castanea*, *P. saturatior* and sheet‐weaver spiders, detecting any trend in population dynamics is complicated.

### (2) Englacial environment

**Fig. 3:**
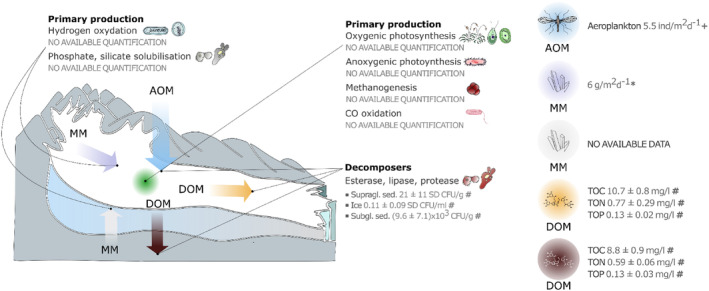
Fluxes of metabolic substrates on the glacier and the metabolic pathways that exploit them. Organisms performing each of the pathways are represented using the same icons as in Fig. 2. AOM, allochtonous organic matter; DOM, dissolved organic matter; MM, mineral matter; supragl. sed, supraglacial sediment; subgl. sed., subglacial sediment; TOC, total organic carbon; TON, total organic nitrogen; TOP, total organic phosphorus. * = debris coverage rate from Azzoni *et al*. (2016); **#** = average colony forming units (CFU) g^−1^ dry mass for sediments, CFU ml^−1^ for ice, and chemical properties of DOM (as TOC, TON, TOP) from Turchetti *et al*. (2008); **+ =** estimated deposition rate of allochthonous aeroplankton, estimated from air traps data from Zawierucha *et al*. (2019*a*).

Englacial habitats encompass gas bubbles, bulk ice, veins of liquid water, englacial channels, and debris within the ice. These environments are linked to the surface by water percolation and may play a role in cycling of organic carbon (Fig. 3). Given the temperate thermal regime of the Forni, we expect the englacial environment to host viable communities (Varliero *et al*., 2021). Current knowledge about biodiversity within the ice of the Forni Glacier is extremely sparse, and reconstructing trophic interactions is impossible. Information on biodiversity in this environment is currently limited to yeasts (Turchetti *et al*., 2008). The bulk ice of the Forni is poorer than meltwater, supraglacial and subglacial sediments both in cell count and species diversity (Turchetti *et al*., 2008), in line with other glaciers studied worldwide (e.g. Butinar, Spencer‐Martins, & Gunde‐Cimerman, 2007). The only species detected in this environment are *Goffeauzyma gilvescens* and *Rhodotorula laryngis*. *G. gilvescens* was also detected in the supraglacial and subglacial environments of the Forni Glacier (Turchetti *et al*., 2008). Its wide distribution within the study site, unique for the detected yeasts, warrants further investigation. In Himalayan sites, *G. gilvescens* showed the ability to solubilise phosphate and silicate (Sanyal *et al*., 2020). These enzymatic activities may be significant in the englacial environment, where the OM contribution from the surface is highly dependent on meltwaters and shows strong seasonal variability. Moreover, the Forni Glacier is surrounded by siliceous rock formations, whose weathering would provide abundant substrates for these enzymes. Such activities would make *G. gilvescens* a primary producer within the englacial habitat. However, these metabolic pathways have not yet been studied on the Forni Glacier. Like other englacial habitats around the world (Boetius *et al*., 2015), bulk ice and ice veins of the Forni Glacier are probably inhabited by bacteria, but no reports are available.

### (3) Subglacial environment

Very little information is available about the subglacial environment of the Forni Glacier. Two studies have investigated the occurrence of yeasts in subglacial sediments (Turchetti *et al*., 2008) and subglacial meltwater flow (Buzzini *et al*., 2005). Subglacial sediments were characterised by a greater number of yeast species than supraglacial debris and ice (Turchetti *et al*., 2008), making it the richest habitat within the Forni in terms of number of yeast species (Turchetti *et al*., 2008). Two species belonging to the genus *Phenoliferia* (formerly *Rhodotorula*; Wang *et al*., 2015) – *P. glacialis* and *P. psychrophenolica* – were detected together with *Leucosporidium creatinivorum* (formerly *R. creatinivora*; de García *et al*., 2015), *Solicoccozyma terricola*, *Goffeauzyma gilvescens*, and *Mrakia gelida*. Many of these species showed positive enzymatic activity for esterase (*G. gilvescens*, *R. laryngis*, *P. glacialis*), lipase (*G. gilvescens*, *P. glacialis*, *R. larynges*), and protease (*M. gelida*, *P. glacialis*, *P. psychrophenolica*) on the Forni and another Alpine glacier (Turchetti *et al*., 2008). No data are available regarding the activity of *L. creatinivorum* on the Forni Glacier. In samples from Antarctica, it has shown activity for esterase, lipase, and protease (Martorell *et al*., 2017). The pool of DOM in glaciers is composed of organic compounds such as lignin, cellulose, proteins, and carbohydrates (Antony *et al*., 2014; Feng *et al*., 2016). Thus, the detected enzymatic activities suggest that these yeasts use DOM as a substrate for at least part of their energy intake. The input of DOM would occur through meltwaters, drained in moulins from the surface to the bedrock, where it circulates through the subglacial drainage system. However, given the high rate of sediment production due to the action of the glacier, it is reasonable to expect mineral matter to be another important substrate in this habitat. Indeed, there is evidence that weathering produces enough H_2_ to sustain the estimated rates of methanogenesis in subglacial habitats – and to be particularly productive where the bedrock is rich in mica (Macdonald *et al*., 2018), as is the case for the Forni Glacier. However, methanogenesis beneath the Forni has not been investigated yet. Bacteria inhabiting anoxic zones in cryoconite holes could seed the subglacial ecosystems through the system of microchannels, streams and moulins as on other glaciers (Zdanowski *et al*., 2017).

## IV. DISCUSSION

The identification of trophic relationships is important in understanding the functioning of an ecosystem. It contributes to highlighting the ecological roles and services provided by different taxa and thus to identifying possible responses to changes in an ecosystem (Schwartz *et al*., 2000; Wagg *et al*., 2019). As shown by our reconstruction of trophic relationships on the Forni Glacier (Fig. 2), (supra)glacial ecosystems require high specialisation and nodes represented by invertebrate species have low redundancy, implying that the disappearance of one species might pose a serious threat to the entire trophic web (Sanders *et al*., 2018). By contrast, the bacterial communities in different habitats of the Forni Glacier are diverse and show redundancy (Franzetti *et al*., 2016, 2017*a*,*b*; Azzoni *et al*., 2018; Pittino *et al*., 2018, 2021). Bacteria take part in both decomposition of OM and primary production, with many bacterial strains supporting similar functions. Therefore, bacterial communities are unlikely to be as fragile as invertebrate communities. In the trophic web of the Forni, specific invertebrate species seem to play a key role in each supraglacial habitat. On the bare ice surface and in coarse debris, ice‐dwelling springtails link the microbial flora to top predators. Ice‐dwelling springtail populations thus appear to be an irreplaceable node in the ice and coarse debris components of the Forni trophic network. By contrast, tardigrades play an important role in cryoconite holes, where they are the apex consumers. Given the importance of these populations, we suggest that further research should focus on determining the intensity of their interactions with other taxa. Indeed, the lack of quantitative information on matter and energy fluxes on this and other glaciers allows only a basic understanding of the interactions within the community. In addition, the roles of springtails and tardigrades on the Forni might be performed by other species on glaciers in different geographical areas. For instance, a key role could be played by ice worms in the Pacific Northwest of the USA (Hotaling *et al*., 2020), by rotifers where they are prevalent (e.g. Zawierucha *et al*., 2021), or by more complex invertebrate communities such as in the Southern Alps of New Zealand (Shain *et al*., 2021). For many areas, there has been little taxonomic classification of glacier‐adapted species, increasing the difficulties of identifying key species.

Glaciers act as cold traps for contaminants, which can promote the selection of bacterial strains with metabolic pathways for their degradation (Cappa *et al*., 2014). Biodegradation of contaminants by bacterial communities has been demonstrated on the Forni (e.g. Ferrario *et al*., 2017). This has implications for the trophic web of the Forni, but also suggests that the ecological communities of glaciers may provide ecosystem services that are yet to be fully investigated. Due to this, the effects of the degraded contaminants on the glacier and down‐valley communities might differ from those of the original compounds. Organisms could still bioaccumulate and biomagnify the non‐degraded contaminants on the ice surface, which may affect their behaviour and metabolism as demonstrated for aquatic and terrestrial arthropods (Gobbi & Lencioni, 2021; Lencioni *et al*., 2023*a*,*b*; Rizzi *et al*., 2022). Buda *et al*. (2024) showed the bioaccumulation of artificial radionuclides in springtails and a link with chlorophyll concentration on glaciers along the Alps, including the Forni Glacier. Undegraded, or partially degraded, contaminants could also be released by meltwaters – damaging downstream ecosystems – or be re‐volatilised. Therefore, reconstructing the metabolic pathways and trophic webs on the glacier surface would help us to assess the fate of such contaminants, potential effects, and hazards also for non‐glacial ecosystems (Beard *et al*., 2022).

The retreat of glaciers will likely cause the extinction of glacier‐specialist taxa (Stibal *et al*., 2020). Species present in peri‐glacial environments (e.g. glacier forelands) will also be affected by the reduced ice mass influence on microclimatic conditions such as temperature, winds, and snow persistence. For example, *Nebria castanea* is already extinct in the forelands of the Forni Glacier (Gobbi *et al*., 2007), while a population is still present on the ice surface (Gobbi *et al*., 2007; Table S1) and in colder forelands of other nearby glaciers (Gobbi *et al*., 2010). Finally, the study of the ecological communities can also provide a better understanding of the influence they may have on glaciological processes and their future development. Dark OM and pigmented supraglacial organisms can reduce glacier albedo and increase ice melting. In turn, the presence of a film of liquid water triggers the growth of organisms, causing a positive bioalbedo feedback loop (Di Mauro *et al*., 2020). However, increased supraglacial runoff can result in the removal of organisms (e.g. Zawierucha, Buda & Nawrot, 2019*c*).

Although the Forni Glacier is a well‐studied site, some information is still lacking: first, in terms of the description and quantification of biodiversity and interactions, and second in terms of habitats. This review has provided a qualitative description of the trophic webs, partly based on the assumption that certain species on the Forni Glacier are likely to perform similarly to their conspecifics in other regions and/or peri‐glacial habitats (such as proglacial plains). Further data are needed to confirm or reject this assumption. Moreover, currently missing information about the biodiversity of englacial and subglacial habitats prevents us from reaching an overview of the trophic relations and biogeochemical cycles in the entire ice body. Mechanisms that could play a role in regulating the bacterial communities and their interactions – such as the presence of viruses – also remain to be investigated on the Forni Glacier. This study aims to lay the foundations for future, more detailed studies that could offer a more comprehensive description of this fragile habitat.

## V. CONCLUSIONS

(1) To the best of our knowledge, this is the first attempt to provide information about glacier biodiversity using a multi‐taxon approach and to reconstruct the trophic web of a glacier. It is essentially a qualitative attempt, partly based on results from other glaciers and adjacent habitats. Further investigations remain needed, for instance using stable isotope analysis of gut contents to provide information on the diet of key organisms.
(2) Due to the rapid retreat of glaciers, their cold‐adapted inhabitants are strongly threatened by climate change. Improving knowledge of the biodiversity and the interactions among various glacier taxa could help predict the critical thresholds of minimum surface and population size beyond which the community will collapse. Such a collapse may in turn impact the surrounding high‐altitude environments that may benefit from the glacier's resources.
(3) To protect taxa adapted to glaciers or to the microclimatic conditions they induce in nearby environments, and to model future changes, it is important to increase our knowledge of trophic relations within glaciers and their links to surrounding environments.

## Supporting information


**Table S1.** Checklist of all taxa detected on the Forni Glacier. (.xlsx)

**Table S2.** Non‐filtered environmental DNA (eDNA) abundance data. (.xlsx).

**Table S3.** Filtered environmental DNA (eDNA) abundance data. (.xlsx).

**Appendix S1.** Filtering methods for eDNA data.

**Table S4.** V9 sequencing abundance data. (.xlsx).

## Data Availability

All additional data described in this review are available at: https://doi.org/10.13130/RD_UNIMI/9PPBAB. Raw demultiplexed data of V9 18S rDNA amplicon sequencing is available under accession numbers SRR28791032 to SRR28791041 of the NCBI project PRJNA1091145.
