# Supplementary material for: Ecological interactions in glacier environments: a review of studies on a model Alpine glacier
Source: Biol Rev Camb Philos Soc. 2024 Sep 9;100(1):227–44. doi: 10.1111/brv.13138 (PMC11718624; doi:10.1111/brv.13138)
Supplement: Supplementary file 4 — Appendix S1. Filtering methods for eDNA data. [file BRV-100-227-s003.docx]

**Appendix S1. Filtering methods for environmental DNA (eDNA) data**

The extracted DNA was amplified with the primers Euka02, which amplifies a ~123 base pair (bp) fragment of 18S rDNA (Guardiola *et al*., 2015; Taberlet *et al*., 2018). This primer pair has very broad taxonomic coverage, including essentially all eukaryotes, and is able to identify the majority of eukaryotes to the family or order level (Guardiola *et al*., 2015; Taberlet *et al*., 2018). In addition to the extraction control, we also amplified two polymerase chain reaction (PCR) controls, containing the PCR mix but no DNA template (Parducci *et al*., 2017). To ensure reliability of 18S results, each sample was analysed in four replicate PCRs, as recommended for eDNA studies on eukaryotes (Ficetola *et al*., 2015, 2019). Sequencing was performed on a Illumina MySeq platform. Obtained DNA sequences were filtered using the OBITOOLS software (Boyer *et al*., 2016), as described in Pansu *et al*. (2015). Sequences were assigned to the relevant taxon using the ecotag program, by comparing them with the European Molecular Biology Laboratory (EMBL) database (Boyer *et al*., 2016).

To ensure reliability of the obtained results, we used the following filtering steps (Zinger *et al*., 2019): we considered a sequence to be present in a PCR replicate if we obtained >3 reads in that replicate. Furthermore, we considered a sequence to be present in a sample if it was detected in at least two PCR replicates performed on that sample. Removing sequences detected in only one PCR replicate allows minimisation of the occurrence of false positives, which can be present for eukaryote eDNA data (Ficetola *et al*., 2015); the minimum number of reads per sample was obtained by inspecting the number of reads in controls (De Barba *et al*., 2014). All sequences detected with >3 reads in more than one PCR replicate of any of the controls was considered to be a potential contaminant and removed from analyses (1.6% of sequences were discarded as potential contaminants). We also removed all human sequences as contaminants.
